# Absence of detection of RSV and influenza during the COVID-19 pandemic in a Brazilian cohort: Likely role of lower transmission in the community

**DOI:** 10.7189/jogh.11.05007

**Published:** 2021-03-01

**Authors:** Fernanda Hammes Varela, Marcelo Comerlato Scotta, Márcia Polese-Bonatto, Ivaine Tais Sauthier Sartor, Charles Francisco Ferreira, Ingrid Rodrigues Fernandes, Gabriela Oliveira Zavaglia, Walquiria Aparecida Ferreira de Almeida, Denise Arakaki-Sanchez, Leonardo Araújo Pinto, Gisele Alsina Nader Bastos, Luiz Antônio Nasi, Maicon Falavigna, Paulo Márcio Pitrez, Renato T Stein, COVIDa study group

**Affiliations:** 1 Social Responsibility – PROADI-SUS, Hospital Moinhos de Vento, Porto Alegre, Brazil; 2 School of Medicine, Pontifícia Universidade Católica do Rio Grande do Sul, Porto Alegre, Brazil; 3 General Coordination, Programa Nacional de Imunização, Brazilian Ministry of Health, Brasilia, Brazil; 4 General Coordination, Programa de Vigilância das Doenças de Transmissão Respiratória de Condições Crônicas, Brazilian Ministry of Health, Brasilia, Brazil

## Abstract

**Background:**

Respiratory syncytial virus (RSV) and influenza are prevalent seasonal community viruses. Although not completely understood, SARS-CoV-2 may have the same means of transmission. Preventive social measures aimed at preventing SARS-CoV-2 spread could impact transmission of other respiratory viruses as well. The aim of this study is to report the detection of RSV and influenza during the period of social distancing due to COVID-19 pandemic in a heavily affected community.

**Methods:**

Prospective study with pediatric and adult populations seeking care for COVID-19-like symptoms during the fall and winter of 2020 at two hospitals in Southern Brazil. RT-PCR tests for SARS-CoV-2, influenza A (Flu A), influenza B (Flu B) and respiratory syncytial virus (RSV) was performed for all participants.

**Results:**

1435 suspected COVID-19 participants (1137 adults, and 298 children). were included between May and August. Median age was 37.7 years (IQR = 29.6-47.7), and 4.92 years (IQR = 1.96-9.53), for the adult and child cohorts, respectively. SARS-CoV-2 was positive in 469 (32.7%) while influenza and RSV were not detected at all.

**Conclusions:**

Measures to reduce SARS-CoV-2 transmission likely exerted a huge impact in the spread of alternate respiratory pathogens. These findings contribute to the knowledge about the dynamics of virus spread. Further, it may be considered for guiding therapeutic choices for these other viruses.

Since December 2019 the world experienced the outbreak of the severe acute respiratory syndrome coronavirus 2 (SARS-CoV-2). SARS-CoV-2 has spread throughout the world with a catastrophic impact not only on public health but also with significant social and economic burden. Brazil is currently the third most affected country, with 9 447 165 confirmed diagnosis of COVID-19 and 230 034 related deaths up to February 7, 2021 [1].

The response to the pandemic has triggered a major change in overall human behavior, with social distancing measures, teleworking, closing of schools and daycare facilities, closing of businesses, strict hygiene behaviors, widespread use of face masks, travel restrictions, and avoidance of activities associated with population gatherings. Brazil has not been an exception to this new scenario, and while it took a while for efficient measures of social distancing to take place, schools and daycare facilities have been closed since mid-March and children have been home since then [2].

As respiratory viruses such as influenza, respiratory syncytial virus (RSV) and SARS-CoV-2 share similar routes and means of transmission, these huge social efforts to prevent the spread of SARS-CoV-2 are also likely to affect the epidemiology of influenza and RSV [3]. Both RSV and influenza have very typical and significant seasonal epidemiology in Brazil, especially during autumn and winter in subtropical areas in the South of the country. An initial concern of health authorities worldwide was the burden of these concomitant infections during the winter months and many clinical guidelines reflected this by indicating both prevention of RSV and treatment of influenza for suspected cases [4,5].

The aim of this study was to report influenza and RSV diagnosis during the SARS-CoV-2 pandemic in pediatric and adult populations with suggestive symptoms of COVID-19 in two health care facilities that serve communities with very different socio-economic backgrounds in the city of Porto Alegre, during local well-defined influenza and RSV seasons [5].

## METHODS

This prospective study was conducted from May 13 to August 31, 2020, in two general hospitals (one private and one public) in Porto Alegre, Southern Brazil, a city whose estimated population was 1 488 252 inhabitants in 2020 [6]. Consecutive adults (>18 year-old) or children older than 2 months were included if presenting at either the outpatient clinics (OPC), emergency departments (ER), or hospitalized with at least one of the following signs or symptoms within 14 days of onset: cough, fever, or sore throat. Exclusion criteria included failure in SARS-CoV-2 sample collection. Clinical and demographic data, as well as samples for viruses testing were collected by a trained research staff at enrollment, according to a standardized protocol.

RT-PCR tests were performed for SARS-CoV-2, influenza A (Flu A), influenza B (Flu B) and RSV for all included subjects. For SARS-CoV-2 analysis nasopharyngeal and oropharyngeal swabs were collected, both swabs allocated in the same transport media. The qualitative Real Time PCR (RT-PCR), was performed with TaqPath^TM^ 1-Step RT-qPCR Master Mix, CG, (Catalog Numbers A15299, AppliedBiosystems, ThermoFisher Scientific, Frederick, USA) and TaqMan^TM^ 2019-nCoV Assay Kit v1 (Catalog Number A47532, ThermoFisher Scientific, Pleasanton, USA), the TaqMan^TM^ 2019-nCoV Control Kit v1 (Catalog Number A47533, ThermoFisher Scientific, Pleasanton, USA) as reaction control. QuantStudio 5 (ThermoFisher Scientific, Waltham, USA) was used to perform protein chain reaction. For RSV, Flu A and Flu B analysis, material from both nostrils were collected with the Xpert®Nasal Sample Collection Swab B/F-100 (Cepheid, Sunnyvale, USA). and allocated in proper transport media (Cepheid, Sunnyvale, USA). Samples were transferred and analyzed in Xpert®Xpress Flu/RSV cartridges (Cepheid, Sunnyvale, USA) [7].

For the statistical analyses, proportions of specimens positive for SARS-CoV-2, influenza, and RSV were stratified by age (ie, children or adults). Normally distributed quantitative data, according to the Shapiro-Wilk test, were expressed as mean ± standard deviation (SD), and non-normally distributed quantitative data were expressed by median and interquartile range (IQR). Categorical variables were described as absolute (n) and relative (%) frequencies.

The study protocol was approved by the Institution’s ethics review board. Written consent was obtained from all participants and/or legal representatives. The study was conducted according to good laboratory practices and in accordance with the Declaration of Helsinki.

## RESULTS

**Figure 1:**
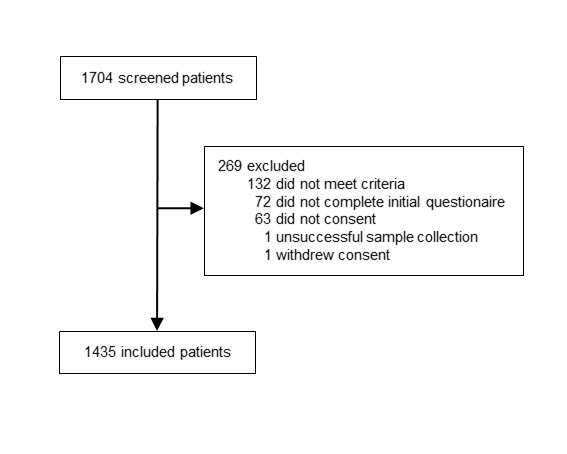
Flow of the participants in the study.

A total of 1435 participants were included in the study from May 13 to August 31, 2020. Of the 1704 screened subjects, 269 were excluded (132 for not meeting inclusion criteria, 63 for not consenting, 72 for not completing initial questionnaire, 1 unsuccessful sample collection, and 1 withdrew consent), as shown in **Figure 1**. Median age for adults was 37.8 years (IQR = 29.6-47.8, range = 18.0-99.9), and for children it was 4.92 years (IQR = 1.96-9.53, range = 0.2-17.3). Median time overall from symptom onset to inclusion was 3 days (IQR = 2-5).

SARS-CoV-2 was detected in 469/1435 (32.7%) participants (420/1137 adults, 36.9%; 49/298 children, 16.4%). Percentage of weekly detection of SARS-CoV-2 varied from 2.2% at the beginning of the inclusion (May) to 54.3% towards the peak (July) of winter in adults, and from 0.0% to 43.8% in children, respectively. 1428 samples from the 1435 (99.5%) were processed for Flu A, Flu B and RSV, and they all resulted negative. Clinical, demographic, and laboratory information about all included patients is summarized in **Table 1****.**

**Table 1:** Clinical, demographic and laboratory characteristics

Variable	Children n = 298	Adult n = 1137
Age (years) – median (IQR)	4.92 (1.97-9.51)	37.79 (29.61-47.77)
Female – n (n%)	157 (52.7)	688 (60.5)
OSS to inclusion (days) – median (IQR)	3.00 (2.00-4.00)	3.00 (2.00-5.00)
Symptoms – n (n%):		
Fever	216 (72.5)	608 (53.5)
Cough	221 (74.2)	891 (78.4)
Loss of smell/taste sensation	157 (52.7)	649 (57.1)
Shortness of breath	118 (39.6)	479 (42.1)
Sore throat	113 (37.9)	738 (64.9)
Headache	131 (44.0)	957 (84.2)
Nausea/vomiting	97 (32.6)	434 (38.2)
Diarrhea	73 (24.5)	376 (33.1)
Conjunctivitis	49 (16.4)	385 (33.9)
Fatigue	123 (41.3)	853 (75.0)
Muscle/joint pain	68 (22.8)	789 (69.4)
Chills	74 (24.8)	648 (57.0)
Comorbidities – n (n%):		
Systemic arterial hypertension	1 (0.3)	190 (16.7)
Diabetes *mellitus*	1 (0.3)	59 (5.2)
Ischemic heart disease	0 (0.0)	17 (1.5)
Cardiac insufficiency	0 (0.0)	18 (1.6)
Chronic obstructive pulmonary disease	3 (1.0)	26 (2.3)
Asthma	52 (17.4)	87 (7.7)
Others	7 (2.3)	21 (1.8)
Laboratory diagnosis:		
SARS-CoV-2	49 (16.4)	420 (36.9)
influenza A	0 (0.0)	0 (0.0)
influenza B	0 (0.0)	0 (0.0)
Respiratory syncytial virus	0 (0.0)	0 (0.0)

n – absolute frequency, % – relative frequency, md – median, IQR – interquartile range (25th and 75th percentiles), OSS – onset signs and symptoms

## DISCUSSION

We have initially expected that COVID-19 frequencies should increase significantly during the fall and winter months, and that the usual patterns of community spread of RSV and influenza A and B would likely change due to public health measures taken to reduce transmission of COVID-19. Interestingly, we have observed a striking absence of these two usually prevalent pathogens in our cohort of symptomatic subjects, despite a detection of 32.7% of SARS-CoV-2. It is important to stress that the region has very well-defined and significant RSV and influenza seasonality yearly [4,5]. Although the hypothesis of lower prevalence for both viruses in this exceptional situation of social distancing was initially reasonable, the observable impact of this reduction is a unique finding.

In USA, Asia and Europe, a number of public health measures aiming to prevent the rapid spread of COVID-19 have started mostly at the end of the winter season. The findings of concomitant viral infections in these communities may have been biased by a natural decline in the incidence of both RSV and influenza, although an unprecedented drop in hospitalization due to RSV has been recently described in Alaska [8]. Wu et al also describe that during the COVID-19 pandemic in China there was a decreasing trend in influenza reports early in 2020, in contrast with two spike waves observed in the previous year [9]. In Switzerland, SARS-CoV-2 almost, and very quickly, completely replaced the seasonally circulating community-acquired respiratory viruses within 3 weeks’ into the pandemic [10]. This finding raises the hypothesis of a possible competition pattern among respiratory viruses. Yet, some reports of substantial rates of viral coinfection make this explanation unlikely [11].

Another possible explanation for lower influenza rates could be heightened awareness due the pandemic, with a subsequent increase in influenza vaccination numbers. However, in Brazil, influenza vaccination rates were 88.8% for the target population, similar to historical values [12]. Furthermore, according to nationwide data from the Brazilian Ministry of Health, from March to August-2020, 643 090 cases diagnosed with severe lower respiratory tract illness (sLRTI) were notified. Among these 52.2% (n=335 748) were positive for SARS-CoV-2 and only 0.4% (2351) for influenza. Similarly, in our region, including Porto Alegre, there were 26 508 notifications of sLRTI; from those 12 717 (48%) were due to COVID-19 and only 43 (0.16%) were positive for influenza for the same time period [13]. These findings are in stark contrast with 2019 data, where the total number of sLRTI cases associated with influenza was 5800 for the whole of Brazil and 388 in our region, considering the same period of time (unpublished data).

The pattern of RSV was similar, with a significant reduction in the number of hospitalized cases with acute viral bronchiolitis throughout the whole country. In our region hospital admissions for infants were 85% lower than in the previous years [14]. Although the local social distancing index varied between 34.2% and almost 60% from March to August, schools and daycare centers remained closed throughout this period. All these measures can be associated with these observed and significant lower levels of transmission for both influenza and RSV. But not good enough to prevent the spread of the highly infectious SARS-CoV-2 virus.

There are a few limitations that may be worth addressing. Data refers to only one city in Brazil at only two health care facilities. Also, subjects were not enrolled using a population-based strategy. However, we believe that the large number of patients in the study, its prospective design, and the inclusion of children and adults from diverse environmental and social backgrounds evaluated throughout usual influenza and RSV seasons outweigh issues of external validity from a convenience sample.

The complete absence of detection of influenza and RSV could raise an issue about the quality of our tests and the specimen collection, still we feel confident with our results. GeneXpert© provides a very automated process, less prone to human processing errors. Moreover, its internal controls (Sample Processing Controls and Probe Check Control) are able to check if there was enough nucleic acid in each sample. We do believe that both influenza and RSV are present in the community, but their numbers are so low that a sample of over 1400 subjects was not able to detect these pathogens.

In summary, our study adds important information regarding the spreading dynamics of high burden respiratory viruses during a period of effective public health measures. The low incidence of RSV and influenza, in contrast with SARS-CoV-2 should be considered in the development of guidelines for antiviral treatment of influenza or in the prevention of RSV with monoclonal antibodies. Moreover, although maintenance such restrictions are not feasible continuously, similar measures could be adopted to control outbreaks due to these viruses. Further, as SARS-CoV-2 prevention depends currently only on non-pharmacological interventions, continuous monitoring of its transmission dynamics is necessary. Hygiene practice and social distancing measures appear to be associated with dramatic reduction of the spread of RSV and influenza.
